# Supplementary material for: Causal Relationship between Aging and Anorexia Nervosa: A White-Matter-Microstructure-Mediated Mendelian Randomization Analysis
Source: Biomedicines. 2024 Aug 16;12(8):1874. doi: 10.3390/biomedicines12081874 (PMC11351342; doi:10.3390/biomedicines12081874)
Supplement: Supplementary file 1 [file biomedicines-12-01874-s001.zip › Supplementary Figure/Figure S4/Singe SNP plot of MR analysis for TL to ubm-b-1570.pdf]

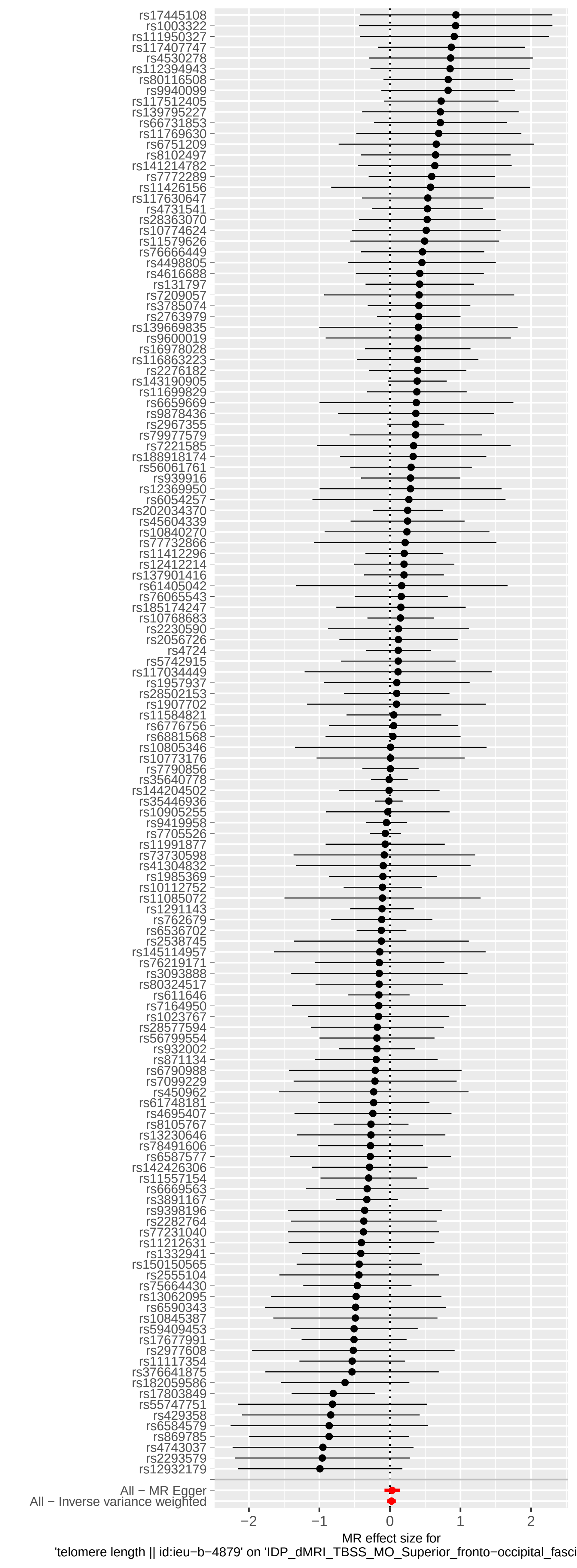
rs17445108
rs1003322
rs111950327
rs117407747
rs4530278
rs112394943
rs80116508
rs9940099
rs117512405
rs139795227
rs66731853
rs11769630
rs6751209
rs8102497
rs141214782
rs7772289
rs11426156
rs117630647
rs4731541
rs28363070
rs10774624
rs11579626
rs76666449
rs4498805
rs4616688
rs131797
rs7209057
rs3785074
rs2763979
rs139669835
rs9600019
rs16978028
rs116863223
rs2276182
rs143190905
rs11699829
rs6659669
rs9878436
rs2967355
rs79977579
rs7221585
rs188918174
rs56061761
rs939916
rs12369950
rs6054257
rs202034370
rs45604339
rs10840270
rs77732866
rs11412296
rs12412214
rs137901416
rs61405042
rs76065543
rs185174247
rs10768683
rs2230590
rs2056726
rs4724
rs5742915
rs117034449
rs1957937
rs28502153
rs1907702
rs11584821
rs6776756
rs6881568
rs10805346
rs10773176
rs7790856
rs35640778
rs144204502
rs35446936
rs10905255
rs9419958
rs7705526
rs11991877
rs73730598
rs41304832
rs1985369
rs10112752
rs11085072
rs1291143
rs762679
rs6536702
rs2538745
rs145114957
rs76219171
rs3093888
rs80324517
rs611646
rs7164950
rs1023767
rs28577594
rs56799554
rs932002
rs871134
rs6790988
rs7099229
rs450962
rs61748181
rs4695407
rs8105767
rs13230646
rs78491606
rs6587577
rs142426306
rs11557154
rs6669563
rs3891167
rs9398196
rs2282764
rs77231040
rs11212631
rs1332941
rs150150565
rs2555104
rs75664430
rs13062095
rs6590343
rs10845387
rs59409453
rs17677991
rs2977608
rs11117354
rs376641875
rs182059586
rs17803849
rs55747751
rs429358
rs6584579
rs869785
rs4743037
rs2293579
rs12932179
All − MR Egger
All − Inverse variance weighted
−2
−1
0
1
2
MR effect size for
'telomere length || id:ieu-b-4879' on 'IDP_dMRI_TBSS_MO_Superior_fronto−occipital_fasci
